# Cystic degeneration of uterine leiomyoma mimicking adnexal mass: a rare clinical image

**DOI:** 10.11604/pamj.2023.44.20.36747

**Published:** 2023-01-11

**Authors:** Cherukuri Srinidhi, Shubhada Jajoo

**Affiliations:** 1 Department of Obstetrics and Gynaecology, Jawaharlal Nehru Medical College, Datta Meghe Institute of Medical Sciences, Sawangi, Wardha, Maharashtra, India

**Keywords:** Leiomyoma, ovarian tumor, cystic degeneration, transabdominal and pelvis sonography

## Image in medicine

Cystic degeneration of uterine leiomyoma is an atypical presentation with an incidence of 4% of all uterine leiomyoma degenerations with risk factors including a family history of uterine leiomyoma, nulliparity, early menarche, late onset of menopause with good prognosis. A 20-year-old girl was accompanied by her parents to the hospital's emergency department with chief complaints of continuous abdominal pain over many months and recent progressive distension of the abdomen. On clinical evaluation, the lump is of 16-week gravid uterus size, occupying the right iliac fossa and hypogastrium with horizontal mobility present, non-tender, regular margins, smooth surface, soft consistency, and no discernible lower border of the mass. Per rectal evaluation revealed a firm, globular mass moving with the cervix. For further investigation, abdomen and pelvis ultrasonography was performed, which showed a large solid cystic mass lesion in the pelvic region with multiple septations measuring approximately 14x11 cm (A) and minimal vascularity on Doppler. The uterus and ovaries could not be visualized separately from the lesion, with the possibility of ovarian tumor etiology followed by a mesothelial cyst, cystic teratoma, endometriotic cyst, and cystic lymphangioma, omental cyst, and mesenteric cysts. Further investigation, like magnetic resonance imaging, couldn't be done due to financial constraints. After giving written and informed consent, the patient underwent laparotomy. Intraoperatively, the finding suggests a uterine mass of approximately 10 x 10 cm in the fundoposterior wall of the uterus. Bilateral fallopian tubes and ovaries are normal. It was intraoperatively diagnosed as leiomyoma of the uterus. A myomectomy was done. About 1 liter of straw-colored fluid oozed from the lesion after the specimen was cut open (B, C). The specimen's histopathological study suggests a fibroid uterus with cystic degeneration.

**Figure 1:**
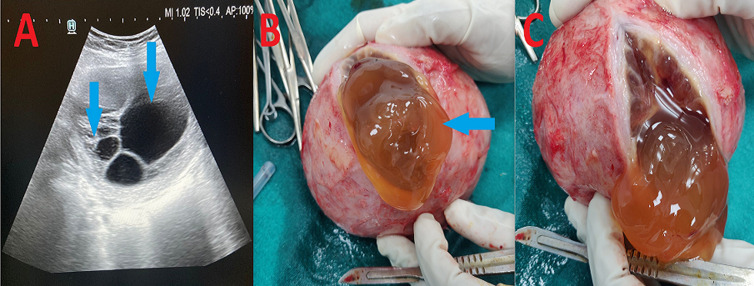
A) a complex cystic mass in the right adnexa with numerous internal septations on transabdominal and pelvis sonography; B, C) cystic degeneration of fibroid with straw-colored fluid oozing out

